# Discretization of the total magnetic field by the nuclear spin bath in fluorine-doped ZnSe

**DOI:** 10.1038/s41467-018-04359-6

**Published:** 2018-05-16

**Authors:** E. A. Zhukov, E. Kirstein, N. E. Kopteva, F. Heisterkamp, I. A. Yugova, V. L. Korenev, D. R. Yakovlev, A. Pawlis, M. Bayer, A. Greilich

**Affiliations:** 1 Experimentelle Physik 2, Technische Universität Dortmund, 44221 Dortmund, Germany; 2 Physical Faculty of St. Petersburg State University, 198504 St. Petersburg, Russia; 3 Spin Optics Laboratory, St. Petersburg State University, 198504 St. Petersburg, Russia; 4 Ioffe Institute, Russian Academy of Sciences, 194021 St. Petersburg, Russia; 5 Peter Grünberg Institute (PGI-9), Forschungszentrum Jülich, 52425 Jülich, Germany; 6 Present Address: Federal Institute for Occupational Safety and Health (BAuA), 44149 Dortmund, Germany

## Abstract

The coherent spin dynamics of fluorine donor-bound electrons in ZnSe induced by pulsed optical excitation is studied in a perpendicular applied magnetic field. The Larmor precession frequency serves as a measure for the total magnetic field exerted onto the electron spins and, surprisingly, does not increase linearly with the applied field, but shows a step-like behavior with pronounced plateaus, given by multiples of the laser repetition rate. This discretization occurs by a feedback mechanism in which the electron spins polarize the nuclear spins, which in turn generate a local Overhauser field adjusting the total magnetic field accordingly. Varying the optical excitation power, we can control the plateaus, in agreement with our theoretical model. From this model, we trace the observed discretization to the optically induced Stark field, which causes the dynamic nuclear polarization.

## Abstract (web summary)

Understanding the electron and nuclear spin interactions is essential to the application of quantum information devices. Here the authors report a step-like electron Larmor frequency versus external magnetic field due to the discretization of the total magnetic field by the nuclear spin bath in ZnSe:F.

## Introduction

Discretization of a physical quantity as a function of a control parameter is of considerable interest, in particular in the quantum regime. Prominent examples are the step-like dependencies that were demonstrated in very different systems: the conductance of a quantum point contact is quantized as a function of channel width with plateaus at integers of 2*e*^2^/*h*^1, 2^; the Hall conductance in a two-dimensional electron gas shows the quantum Hall effect with steps in units of *e*^2^/*h* as a function of magnetic field^3^; the ac-Josephson effect demonstrates quantized voltage steps (Shapiro steps) given by multiples of *ħω*/2*e* in a microwave field of frequency *ω*^4, 5^. Here *e* is the electron charge and *ħ* = *h*/2*π* is the reduced Planck constant. In a many-body system, the discretization is typically the consequence of a feedback mechanism, which drives the system into a particular preferential state and stabilizes it there.

We report another example of discretization, based on the feedback between the electron spins and the nuclear spin bath in ZnSe doped with fluorine, which is exposed to an applied magnetic field. As a result of this feedback, the total magnetic field contributed by the applied and local nuclear field and assessed through the electron spin precession frequency, becomes discretized:

1 $$B_k = kB_0 = k \times \frac{h}{{g_{\mathrm{e}}\mu _{\mathrm{B}}}} \times \frac{1}{{T_{\mathrm{R}}}},$$

where *k* is an integer. In the field unit *B*_0_, *μ*_B_ is the Bohr magneton and *g*_e_ is the Lande factor of the electron spins that are periodically excited by laser pulses separated in time by *T*_R_.

The localization of an electron spin in a nanostructure leads to a strong coupling to the nuclear spin bath within the volume of the electron wave function. The nuclear bath acts in effect as a fluctuating local magnetic field, which typically leads to accelerated dephasing of the electron spin coherence. This obstacle may be overcome by either preparing a host material composed of zero nuclear spin isotopes^6–9^ or by suppressing the random nuclear fields. Two strategies were suggested for the second approach: (i) polarizing the nuclear spins almost entirely up to 100%^10^ and (ii) using the strong feedback in the electron-nuclear system for fluctuation suppression.

While strategy (i) so far has remained elusive for systems with many nuclear spins, several groups achieved locking of the nuclear field to a fixed, yet undefined value and demonstrated narrowing of the nuclear field distribution. Reference^11^ showed resonant nuclear locking driven by electron-nuclear coupling under microwave irradiation, while in refs.^12, 13^ locking to the optical transition energy resonantly addressed by a laser was used. An increase of the spin coherence through a narrowing of the nuclear field distribution was reported for electrically controlled double quantum dots (QDs)^14, 15^ and optically excited ensembles of singly charged QDs^16, 17^. The strong electron-nuclear feedback in singly charged (In,Ga)As/GaAs QDs leads to nuclear-induced frequency focusing^16^. The inhomogeneous distribution of electron precession frequencies about a magnetic field is focused to a few modes given by multiples of the laser repetition rate by adjusting the nuclear spin polarization in each QD correspondingly. Therefore, the nuclear spins whose action is typically considered as destructive, act constructively by locking the electron spin precession in the QD ensemble to these modes. Still, the strength of the nuclear field is adjusted individually in each dot and, therefore, shows a large scatter.

In this paper, we identify a model system in which the nuclear spin bath leads to a prominent focusing effect; the nuclei collaborate such that the total magnetic field acting on the fluorine donor-bound electron spins in a ZnSe host matrix becomes discretized in units of the effective magnetic field *B*_0_ as defined by Eq. (1). This is evidenced by the fact that the electron spin precession frequency shows a step-like behavior when tuning the applied magnetic field. We also show that the discretization can be changed by the excitation power level. Our calculations reveal that a power level change corresponds to a change of optical detuning from the transition frequency, which can lead to a focusing of the electron spins to a different precession frequency.

## Results

**Fig. 1:**
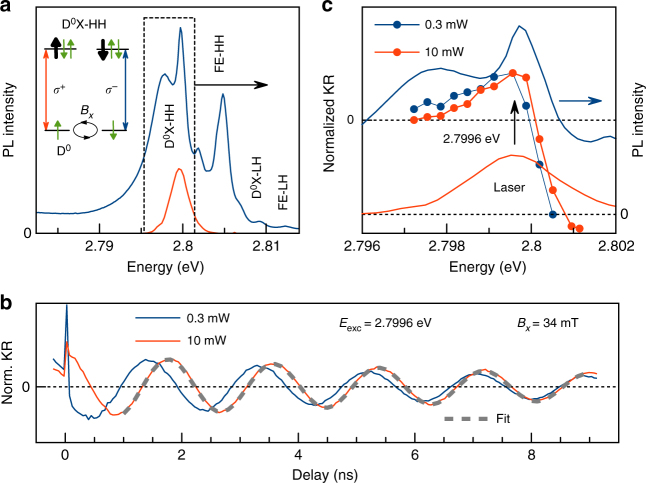
Kerr rotation at the heavy hole neutral donor transiton. **a** Normalized PL spectrum of the studied sample. Red line is the laser spectrum. Sketch on the left represents the level scheme of total energy states for the single electron ground state D^0^ (thin green arrow) and an excited state composed from a two-electron singlet state and a heavy hole (thick black arrow). *B*_*x*_ is the applied magnetic field, and *σ*^±^ indicates the circularly polarized laser excitation with corresponding selection rules. **b** Normalized Kerr rotation signal measured for resonant D^0^X-HH excitation at *B*_*x*_ = 34 mT for two pump powers, 0.3 and 10 mW. Gray dashed line is a fit to the high power data. **c** Spectral dependence of the normalized Kerr rotation amplitude at −50 ps delay for 0.3 mW (blue) and for 10 mW (red). *T* = 1.8 K

**Experimental observation of discretization**. Figure 1a shows the normalized photoluminescence (PL) spectrum of the ZnSe:F sample, measured under continuous-wave excitation with a photon energy of 3.05 eV. Besides the free excitons (FE) of the heavy hole (HH) and light hole (LH), the corresponding neutral donor-bound excitons D^0^X can also be seen. In the applied transverse magnetic field, the ground (D^0^) and excited (D^0^X) states are split by the Zeeman energy. The D^0^ electron spin 1/2 splits into two states. The splitting of the D^0^X state is defined solely by the hole spin. Due to confinement and strain in the structure, only the low energy HH states with spin ±3/2 are considered^18, 19^. The schematic in Fig. 1a presents the corresponding optical selection rules in the *z*-basis^20^. For further details on the sample structure and a complete description of the PL spectrum, we refer to ref.^21^. Figure 1b shows normalized time-resolved Kerr rotation (TRKR) signals for low (0.3 mW) and high (10 mW) pump power, measured in the Voigt geometry for *B*_*x*_ = 34 mT, oriented normal to the optical axis taken as *z*-axis. The spin ensemble is oriented by the pump pulses arriving at time zero along this *z*-axis and then precesses in the *zy*-plane around the applied magnetic field along the *x*-axis. Figure 1b shows an exemplary fit to the high power data (the dashed gray line) in the delay time range 1–9 ns using the form: $A_{{\mathrm{KR}}}$ = $A_0\,{\mathrm{cos}}(\omega t + \varphi ){\mathrm{exp}}\left( { - t{\mathrm{/}}T_2^ \ast } \right)$, with the initial electron spin polarization *A*_0_, the pump-probe delay *t*, the oscillation phase *φ* and the spin dephasing time $T_2^ \ast$. In this case $T_2^ \ast = (10.0 \pm 0.2)$ ns. The dephasing times at low magnetic fields, as used in this work, are limited by the nuclear spin fluctuations in the donor surroundings, see ref.^21^. The main feature in Fig. 1b is the significant difference in the electron spin precession frequencies of *ω* (*P* = 10 mW) − *ω* (*P* = 0.3 mW) = 3.499 − 3.362 = 0.137 ns^−1^ for the two used powers. In Fig. 1c we show the photon energy dependencies of the normalized Kerr rotation amplitude throughout the excited donor states for both pump powers. The implications of the relative energy shift between the two dependencies are discussed later in the paper.

**Fig. 2:**
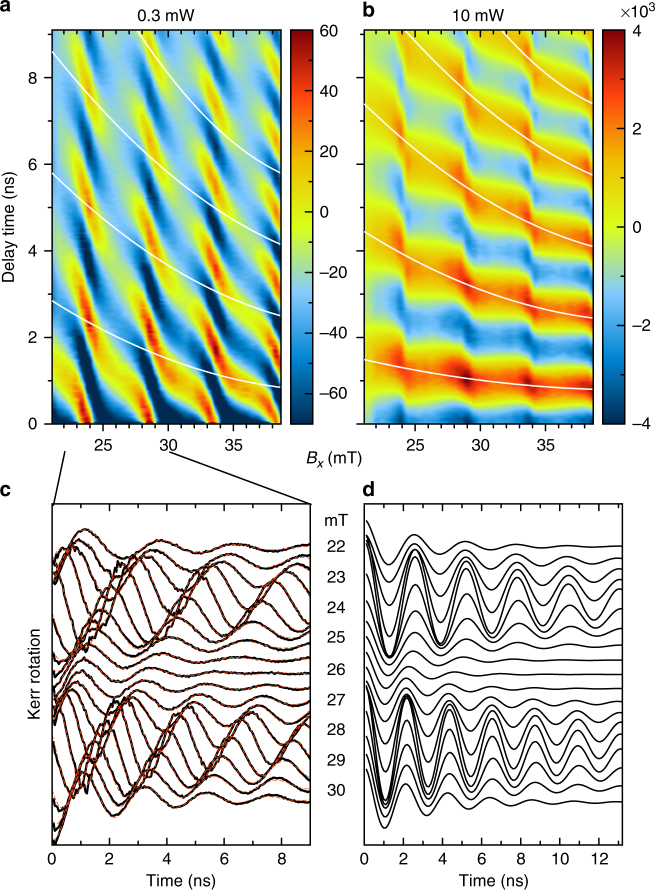
Magnetic field dependence of time-resolved traces. Contour plots of TRKR signal as function of applied magnetic field *B*_*x*_ and delay time, using pump excitation powers of 0.3 mW (**a**) and 10 mW (**b**). Note the different intensity scales in the two panels. White lines are the calculated magnetic field dependencies of the electron Larmor frequency without nuclear contribution. **c** Waterfall of the TRKR spectra in the magnetic field range of 22–30 mT (black lines). Red dashed lines, plotted over the black lines, are the fits by *A*_KR_. **d** Extracted fit functions from **c** are shown with phase *φ* set to zero to highlight the stability of the frequencies around a whole integer of *T*_R_

To obtain insight into the precession frequency variation, we perform TRKR measurements in varying magnetic field. In detail, we measure the TRKR in the field range from 20 to 40 mT in steps of 0.5 mT. A similar behavior is observed in a higher magnetic field from 56 to 64 mT, see Supplementary Note 2. Figure 2 shows contour plots of TRKR vs. applied magnetic field *B*_*x*_ and delay time between pump and probe, measured for 0.3 mW (a) and 10 mW (b) pump powers. One expects a linear scaling of the precession frequency with the magnetic field *B*_*x*_ according to *ω*_e_ = *g*_e_*μ*_B_*B*_*x*_/*ħ*, with *g*_e_ = 1.13. This *g*_e_ value was determined from the mean dependence over a large range of magnetic fields. Accordingly, one would expect in these contour plots that the oscillation period along the temporal axis shortens smoothly with increasing field, as shown by the white lines. The two contour plots show a different behavior. For high power, the smooth evolution from low to high fields is superimposed by significant discontinuities occurring over relatively small field ranges around 24, 29, and 34 mT. The behavior is even more pronounced at lower power where in some field ranges the oscillation period is constant. However, the contour plot is distorted as the plateaus appear strongly tilted through the phase variation of the Kerr rotation signal in the magnetic field. To demonstrate the frequency stability at low power in a more explicit way, we show the waterfall diagram of the spectra in the magnetic field range 20–30 mT together with corresponding fit functions, see Fig. 2c. Further, Fig. 2d shows the extracted fit functions with the phase fixed to zero, plotted over the whole pulse repetition period *T*_R_. These curves demonstrate the frequency stability over the ranges of magnetic fields with the integer number of Larmor precession periods being 5 in the range of about 22–25 mT and 6 in the range 27–30 mT. Additionally, the strong variation of the dephasing time with the magnetic field is observed. It changes between 10 ns at the plateau centers and about 2 ns in the transition regions between the plateaus. In what follows, we concentrate on the analyses of the frequencies.

**Fig. 3:**
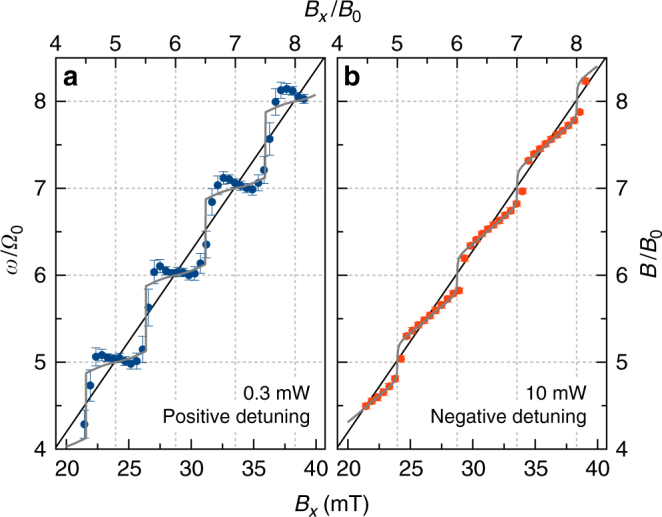
Plateaus in precession frequency vs. magnetic field. Dependence of precession frequency *ω* (given in units of laser repetition rate) on applied field in absolute units (bottom) and units of *B*_0_ (top). The precession frequency can be converted into the total magnetic field inside the sample by $B = \hbar \omega {\mathrm{/}}\left( {g_{\mathrm{e}}\mu _{\mathrm{B}}} \right)$. The total magnetic field is given on the right axis in units of *B*_0_. The symbols show the experimental data; the gray lines are calculations according to our model with: *T*_2_ = 5*T*_R_, $\Theta = 1.2\pi$, *A*_Se_ = 33.6 μeV, *I*_Se_ = 0.5. For low pump power we use *Δ* = −0.16 meV as detuning, while for high pump power we use *Δ* = 0.03 meV. Black lines are the expected electron Larmor frequency *ω*_e_ for *g*_e_ = 1.13. Error bars define a standard deviation in the frequency fit

From analyzing the plots with the fit function *A*_KR_, one can extract the precession frequency, as shown in Fig. 3 in units of the laser repetition frequency *Ω*_0_ = 2*π*/*T*_R_ (left axes), vs. the applied field *B*_*x*_ in absolute units (bottom) and in units of *B*_0_ (top), confirming the surprising finding of strong deviations from the linear dependence (the solid black line) on *B*_*x*_: the precession frequency shows a non-monotonous behavior with distinct steps. The steps are most prominent for the low power case (blue points in Fig. 3a), where pronounced plateaus are seen, separated from each other by *Ω*_0_, so that the precession frequencies are given by *ω* = *kΩ*_0_ with integer *k*. The plateaus are centered around *B*_*x*_ = *kB*_0_ with the same *k* and the jumps from a plateau to the next higher one, *k* + 1, occur at the field strengths *B*_*x*_ = (2*k* + 1)*B*_0_/2. The equation *ω* = *kΩ*_0_ is identical to the previously observed mode-locking condition in a QD ensemble, where the large inhomogeneity in the ensemble prevented the observation of precession frequency discretization^17^. Turning to the high pump power case in Fig. 3b, one still finds indications of the discretization even though the steps are somewhat tilted and smoothed. Furthermore, the staircase is shifted now: the plateaus have an average frequency of *ω* = (2*k* + 1)*Ω*_0_/2, and the jumps between them occur at *B*_*x*_ = *kB*_0_. Taking the *B*_*x*_-linear frequency as a baseline, the measured precession frequency shows symmetric, quasi-oscillatory behavior around it, with sections with comparatively enhanced frequency alternating with sections of reduced frequency.

**Fig. 4:**
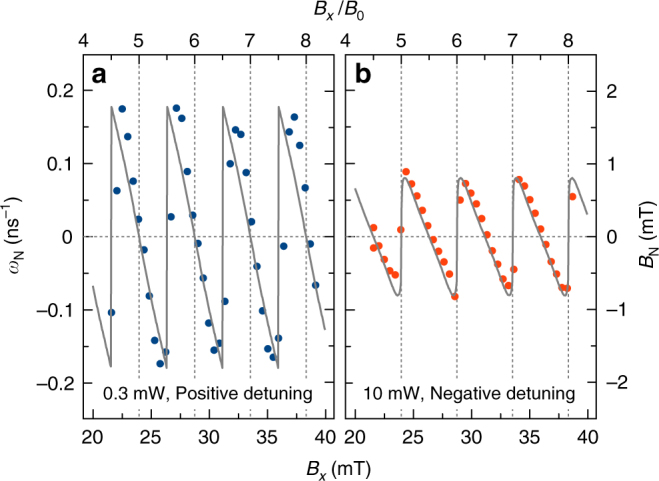
Magnetic field dependence of the local field. Deviation *ω*_N_ of the electron spin precession frequency *ω* from the expected linear dependence $\omega _{\mathrm{e}} = g_{\mathrm{e}}\mu _{\mathrm{B}}B_x{\mathrm{/}}\hbar$ (left) and corresponding Overhauser field *B*_N_ (right) as function of *B*_*x*_ (bottom axis) and *B*_*x*/_*B*_0_ (top). **a** shows the low pump power case, **b** the high power case. Symbols give the experimental data and the gray lines are calculations with the same parameters as in Fig. 3

The precession frequency is determined by the total magnetic field experienced by the electron spins, *B* = *ħω*/(*g*_e_*μ*_B_). This total field *B*, therefore, shows the same discretization as the precession frequency, see the right axis in Fig. 3. The deviations of *B* from the applied field *B*_*x*_ demonstrate the build-up of an additional magnetic field with the same orientation that adds to or subtracts from *B*_*x*_. The only possible origin for the additional magnetic field is the nuclear spin bath around each fluorine donor^16^. To evaluate this local nuclear-induced field we first calculate *ω*_N_ as the difference between the measured frequency *ω* and the precession frequency determined solely by the applied field *ω*_e_: *ω*_N_ = *ω* − *ω*_e_. This frequency is shown on the left axis of Fig. 4a, b as function of *B*_*x*_ (bottom) and *B*_*x*_/*B*_0_ (top). *ω*_N_ can be converted into the nuclei-induced magnetic field by *B*_N_ = *ħω*_N_/(*g*_e_*μ*_B_), given by the right axis. Both, the frequency *ω*_N_ and the magnetic field *B*_N_ show a periodic, saw-tooth-like variation around zero. Strikingly, the dependencies at high and low pump power are shifted in phase by *π*/2. For positive (negative) *B*_N_, the applied field is amplified (attenuated), thereby increasing (decreasing) the precession frequency of the electron spins, so that they fulfill the mode-locking condition through the discretized magnetic field.

**Fig. 5:**
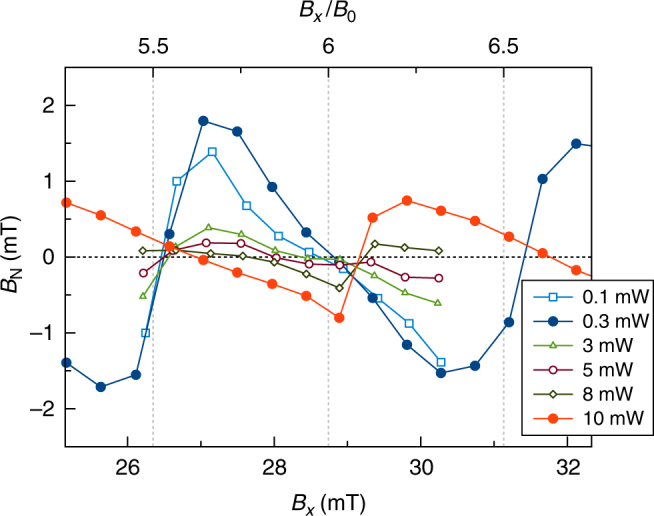
Power dependence of the local field. Laser power dependence of induced Overhauser field (*B*_N_) on the applied magnetic field (*B*_*x*_) in absolute units (bottom) and units of *B*_0_ (top). It demonstrates a smooth transition between positive and negative detuning that is controlled by the laser power

In all presented experiments, we have shown only the two limiting cases for the laser powers: 0.3 and 10 mW. Figure 5 demonstrates the laser power dependence of the induced Overhauser field vs. the applied magnetic field. Starting from low powers, the Overhauser field increases with laser power reaching the value of *B*_N_ = 1.8 mT for 0.3 mW power. With further increase, the induced nuclear field smoothly decreases down to zero and switches its sign for powers around 5 mW. The maximal pump power of 10 mW is in our case a compromise dictated by the experimental conditions. At higher powers, the sample starts to be strongly affected by the laser excitation, which leads to accelerated spin dephasing and relaxation^21^.

### Theory

As mentioned in the introduction, there is a variety of investigations describing the observation of the nuclear field locking and dragging^22^. To support this variety of experiments, several principally different models were proposed. In ref.^23^ the authors suggest that the central mechanism responsible for the locking and dragging effects is related to the noncollinear hyperfine interaction between the nuclear spins and the HH spin. The strength of this effect relies on mixing of HHs and LHs. Further experiments, provided on strained self-assembled QDs presented in refs.^12, 24^, rely on an alternative origin of the noncollinear interaction, which can be induced by large quadrupolar fields. Furthermore, dragging can be provided by the optical Stark effect^25^ or the inverse Faraday effect^26^, where the polarization direction of nuclei is controlled by the optical detuning between the laser photon energy and the optical transition of a trion state. Depending on the studied system, these effects might have different contributions.

A special class of locking phenomena is obtained by experiments with pulsed-laser excitation, where the carrier Larmor frequency in a transverse magnetic field becomes equal to harmonics of the periodic pulsed laser excitation. In ref. ^16^ the authors suggest the strong modulation of the nuclear relaxation time in singly charged QDs to be responsible for this synchronization. The noncollinear hyperfine interaction drives the nuclear spin diffusion under pulsed-laser excitation, providing a variation of the nuclear environment in each QD^16, 27^. A further experiment demonstrated an efficient and directed locking process using the optical Stark effect together with strong modulation of the nuclear spin relaxation to explain the observations^28^, while the model presented in ref. ^29^ suggested that the optical Stark effect alone is sufficient. Several complementary theoretical models were proposed to explain the observed locking effects for pulsed periodical excitation, see refs.^30–34^.

Let us now consider our experimental findings theoretically. The strain effects in the present structure lead to a clear energy separation of the LH and HH states. It allows us to base our consideration on the well-studied optical transition introducing a photogenerated HH exciton coupled to a single donor-bounded electron, similar to self-assembled QDs^18^, see Fig. 1a. Additional simplification is provided by the fact that the surrounding nuclei do not have quadrupole moments, due to the nuclear spin 1/2 for ^77^Se. Furthermore, the nuclear spin diffusion is suppressed in the sample due to the inhomogeneous Knight field leading to inhomogeneous nuclear polarization in the donor surrounding^35^.

Circularly polarized pump pulses create and support the electron spin polarization in the TRKR experiments. The synchronization condition of the laser repetition frequency *Ω*_0_ with the Larmor precession frequency *ω*_e_ is important at this point as it creates optical orientation of the ensemble, whose average spin is close to the maximal value of 1/2 and rotates with a frequency *ω*_e_ = *kΩ*_0_ around the field *B*_*x*_^36^. However, the projection of the mean spin *S*_*x*_ on the field *B*_*x*_ is zero. In order to have *S*_*x*_ ≠ 0, a small deviation from the exact synchronism is necessary.

Furthermore, when spectrally detuned from the donor optical transition, the optical pulses cause an energy splitting between the two-electron spin eigenstates. This effect can be described as an effective magnetic field along the optical wave vector (*z*-axis), the optical Stark field^20, 25^. When exposed to this effective field, the electron spin becomes rotated around the *z*-axis. Then the joint action of the Stark field and the applied magnetic field leads to a deviation of the mean spin from the *z*-axis and a non-zero projection of *S*_*x*_.

To be more specific, the action of a short, circularly polarized optical pulse on an electron spin system is given by^37^:

2 $$S_x^ + = Q\,{\mathrm{cos}}\,{{\Phi }}S_x^ - + Q\,{\mathrm{sin}}\,{{\Phi }}S_y^ - ,$$

3 $$S_y^ + = Q{\mathrm{cos}}\,{{\Phi }}\,S_y^ - - Q\,{\mathrm{sin}}\,{{\Phi }}\,S_x^ - ,$$

4 $$S_z^ + = \frac{{Q^2 - 1}}{4} + \frac{{Q^2 + 1}}{2}S_z^ - ,$$

where $S_i^ +$ and $S_i^ -$ are the spin polarizations along the axis *i* before (−) and after (+) the pump pulse. *Q* and *Φ* are characterizing the pulse. For a square pulse they are given by:

5 $$Q = \sqrt {1 - \frac{{{{\Theta }}^2}}{{{{\Omega }}_{\mathrm{R}}^2}}\mathop {{{\mathrm{sin}}}}\nolimits^2 \frac{{{{\Omega }}_{\mathrm{R}}}}{2}} ,{{\Phi }} = \pi {{\Delta }} - \phi ,$$

with the area ${{\Theta }} = {\int} {2\left| {\left\langle d \right\rangle E(t)} \right|dt{\mathrm{/}}\hbar}$ of the pulse, determined by the transition matrix dipole element $\left\langle d \right\rangle$ times the electric field envelope *E*(*t*) of the pulse with duration *τ*_p_; ${{\Omega }}_{\mathrm{R}} = \sqrt {(2\pi {{\Delta }})^2 + {{\Theta }}^2}$ is the Rabi frequency; *Δ* = (*ω*_p_ − *ω*_t_)*τ*_p_/(2*π*) is the detuning of the optical laser frequency *ω*_p_ from the optical transition frequency *ω*_t_, and sin *ϕ* = (2*πΔ*/(*QΩ*_R_)) sin(*Ω*_R_/2).

Taking into account the spin dynamics in magnetic field averaged over the laser repetition time *T*_R_ in an infinite series of laser pulses, allows us to calculate the mean 〈*S*_*x*_〉 component^37^:

6 $$\left\langle {S_x} \right\rangle = K\frac{{1 - Q^2}}{{4C}}\frac{{T_2}}{{T_{\mathrm{R}}}}\left( {1 - e^{ - T_{\mathrm{R}}/T_2}} \right){\mathrm{sin}}\left( {\omega T_{\mathrm{R}}} \right),$$

where *T*_2_ is the single spin coherence time and *K* and *C* are defined by Eq. (30) in ref.^37^. Here, *K* is changing its sign depending on detuning *Δ*.

Further, nonlinearity is included: for dynamic nuclear polarization, the created electron spin component *S*_*x*_ is transferred by a flip-flop process to the nuclear system^29^:

7 $$\frac{{{\mathrm{d}}I_{\mathrm{N}}}}{{{\mathrm{dt}}}} = - \frac{1}{{T_{{\mathrm{1e}}}}}\left[ {I_{\mathrm{N}} - \bar Q\left\langle {S_x(I_{\mathrm{N}})} \right\rangle} \right] - \frac{{I_{\mathrm{N}}}}{{T_{{\mathrm{1L}}}}},$$

where *I*_N_ is the average nuclear spin polarization, *T*_1e_ is the nuclear relaxation time due to interaction with electrons, and *T*_1L_ is the nuclear spin-lattice relaxation time, which comprises any other possible leakage mechanism; $\bar Q = 4I(I + 1){/}3 = 1$ for *I* = 1/2 (the spin of ^77^Se). The nuclear spin polarization creates an effective Overhauser field, which changes the electron spin precession frequency *ω*_e_ in the applied magnetic field *B*_*x*_:

8 $$\omega = \omega _{\mathrm{e}} + \omega _{\mathrm{N}},$$

where *ω*_N_ = *AI*_N_*χ*/*ħ*, with the hyperfine interaction constant *A* and the isotope abundance *χ*.

The optical Stark field, and therefore the nuclear spin polarization, depend on the sign of the detuning between laser and optical transition, as *Φ* in Eq. (5) changes its sign with reversal of the detuning *Δ*. For positive detuning $\left( {E_{{\mathrm{pump}}} > E_{{\mathrm{D}}^{\mathrm{0}}{\mathrm{X}}}} \right)$, *S*_*x*_ < 0 and *S*_*y*_ > 0, so that the average nuclear spin is oriented along the applied magnetic field in the field range (*k* − 1/2)*B*_0_ ≤ *B* ≤ *kB*_0_, which increases the precession frequency such that a plateau *kΩ*_0_ is reached. On the other hand, the nuclear spin is oriented opposite to *B*_*x*_ in the range *kB*_0_ ≤ *B* ≤ (*k* + 1/2)*B*_0_. For negative detuning, *S*_*x*_ > 0 and *S*_*y*_ > 0, so that the sign of nuclear spin polarization is inverted. This has consequences for the plateaus of the total magnetic field and the precession frequency: for positive detuning the precession frequency is tuned to integer modes of the laser repetition rate, so that the total magnetic field is a multiple of the field quantum *B*_0_, while for negative detuning the plateaus occur just in between these integer values at half integers of *Ω*_0_ and *B*_0_, see Fig. 3.

Based on the presented theory, it is impossible to change the direction of the nuclear spin polarization purely by the pump power, see also Supplementary Note 1. In experiments, however, we see that the sign and the value of nuclear polarization are controlled by the applied laser power, see Fig. 5. Our model reproduces the experimental behavior by varying the laser power and detuning values. The results of the calculations reproduce data quite well if one uses the following correspondence with the experiment: positive detuning corresponds to low power case, while negative to high power. Indications for this detuning change are provided in Fig. 1c by the spectral dependence of the TRKR amplitude in the D^0^X-HH vicinity for the two studied pump powers (red and blue points). The amplitude is taken before the pump pulse arrival at *t* = −50 ps and demonstrates an energy shift of 0.15 meV between low and high pump power, that may be related to the change of detuning that also inverts its sign. The origin of the shift may be related to the variation of the carrier population at increased pump power, shifting the absorption profile to higher energies relative to the laser spectrum. Furthermore, to take the ensemble effects into account, we have integrated the detuning *Δ* over a finite range. In Fig. 3b for negative detuning of −0.16 meV, *Δ* is integrated over −0.159 ÷ −0.161 meV, while for positive detuning of 0.03 meV, *Δ* is integrated over 0.029 ÷ 0.031 meV, in order to obtain best agreement. For a model dependence of Overhauser field on different parameters see Supplementary Note 1. Additionally, Supplementary Notes 3 and 4 provide dependencies of the Overhauser field on electron concentration and temperature, respectively.

## Discussion

The calculations presented in Fig. 3 by the gray lines, are in good accord with the data. This is also the case for the applied magnetic field dependence of the frequency difference and the underlying Overhauser field that is driven by the effective optical Stark field of the laser pulses, see Fig. 4. From the Overhauser field, we can estimate the nuclear spin polarization *I*_N_. We take the low power case as an example. ^77^Se has a nuclear spin *I* = 1/2 with an abundance *χ* = 7.58%, while ^67^Zn has *I* = 5/2 and *χ* = 4.11%. The corresponding hyperfine constants are *A*_Se_ = 33.6 μeV and *A*_Zn_ = 3.7 μeV^21^. This strong difference in the hyperfine coupling allows us to focus on the selenium nuclei and neglect the zinc nuclei. This assumption is confirmed by nuclear depolarization experiments applying additional radio-frequency fields^35, 38^. Pumping the zinc nuclei has no impact on the observations. From *ω*_N_ = *A*_Se_*I*_N_*χ*/*ħ* = 0.18 ns^−1^, we obtain *I*_N_ = 0.046, corresponding to 4.6% of the maximum achievable nuclear polarization. According to ref.^29^ the build-up of average nuclear spin polarization should lead to a hysteresis effect, see Supplementary Note 1. As a consequence, the frequency stabilization ranges would be extended over the mode separation *Ω*_0_ and would depend on the scan direction of the field. Our experiments do not reveal hysteresis.

We emphasize, however, that the presented theory does not take into account the intrinsic nuclear spin fluctuations, which destabilize the nuclear polarization for reduced electron-nuclear feedback strength. An alternative explanation of the discretization that does not rely on the increasing average nuclear spin, but on a reconfiguration of the nuclear fluctuations was given in ref.^33^. The nuclear fluctuation field in the studied sample is about *B*_f_ = 1.65 mT^21^, while the maximal Overhauser field is about *B*_N_ = 1.8 mT, see Fig. 4. On the other hand, following ref.^30^ the nuclear fluctuations should be reduced in the middle of the plateaus due to the strong electron-nuclear feedback, see Supplementary Note 5. This allows for an application of the average spin concept in carrying out a semi-classical description. At the position between the plateaus, the feedback is reduced, and the nuclear polarization becomes unstable with respect to other precession modes, so that the total magnetic field and therefore the precession frequency switches to a different plateau. A full microscopic theory would require the inclusion of the spin fluctuations in a quantum-mechanical many-body description to explain the quantization of the total magnetic field. Also, aspects like the optical excitation and subsequent statistical decay, in general, require a quantum-mechanical description beyond our effective model.

This approach can be extended to other semiconductor systems having localized magnetic moments (electrons or holes) interacting with the nuclear surrounding. Moreover, the observed effect can be expected in the wide class of diluted magnetic semiconductors, where the localized spins of magnetic ions, e.g., Mn^2+^ in (Cd,Mn)Te or (Zn, Mn)Se, have strong exchange interaction with the carrier spins. In this case, the Mn spins take over the role of the nuclear spins, and one could exploit the exchange coupling instead of the much weaker hyperfine interaction. Furthermore, any spin system based on paramagnetic impurities with incomplete *d*− and *f*− shell located within the localization volume of D^0^X complex is a good candidate for inducing precession frequency discretization.

In conclusion, we have presented a system based on donors in ZnSe with reduced inhomogeneity compared to QDs in which the fluorine donor-bound electron spins experience a discretized total magnetic field, when the applied field is ramped, as evidenced by the Larmor frequency showing a step-like dependence. The discretization is enabled by the action of the nuclear spin bath with a suitably adjusted polarization component along the applied magnetic field due to the optical Stark field of the laser pulses. The discretization may be controlled by a precise adjustment of the detuning between optical transition and laser photon energy controlled by the laser power. The underlying electron-nuclear feedback should also lead to a narrowing of the nuclear fluctuations, which is expected to lead to a prolongation of the inhomogeneous electron spin dephasing time $T_2^ \ast$. Using advanced pump-probe schemes for measuring long-lasting spin coherence, see ref. ^39^ one may be able to assess the prolongation quantitatively.

## Methods

### Sample structure

We study a homogeneously doped 70-nm-thick ZnSe epilayer with fluorine donor concentration of about 10^15^ cm^−3 ^^21^, grown by molecular-beam epitaxy on top of a 20-nm-thick Zn_0.92_Mg_0.08_Se layer, which prevents carrier diffusion into the GaAs substrate with a smaller band gap. The sample is placed in an optical cryostat with a superconducting split-coil magnet. The sample temperature is *T* = 1.8 K for all measurements.

### Experimental setup

We use pump-probe TRKR to study the dynamics of the donor-bound electron spins^40, 41^. Electron spin coherence is generated by a train of circularly polarized pump pulses of 1.5 ps duration emitted by a mode-locked Ti:Sapphire laser operating at a repetition period *T*_R_ = 13.2 ns. The excited spin coherence is measured by the Kerr rotation of the linearly-polarized probe pulses. The pump power is varied, while the probe power is fixed at 0.5 mW in all measurements. The spot diameters on the sample are 300 μm. The central photon energy is tuned into resonance with D^0^X-HH at 2.7996 eV (442.87 nm), see the red line in Fig. 1a, for which the laser beam is frequency-doubled by transmission through a Beta-Barium Borate crystal. The half width at half maximum of the laser is about 1 meV compared to 4.4 meV width of the D^0^X-HH transition. The pump helicity is modulated at *f*_m_ = 235 kHz between *σ*^+^ and *σ*^−^ polarization using an electro-optical modulator, while the probe is kept unmodulated.

### Data availability

The data that support the findings of this study are available from the corresponding author upon reasonable request.

## Electronic supplementary material

Supplementary Information
