# Supplementary material for: Discretization of the total magnetic field by the nuclear spin bath in fluorine-doped ZnSe
Source: Nat Commun. 2018 May 16;9:1941. doi: 10.1038/s41467-018-04359-6 (PMC5955946; doi:10.1038/s41467-018-04359-6)
Supplement: Supplementary file 1 — Supplementary Information [file 41467_2018_4359_MOESM1_ESM.pdf]

Discretization of the total magnetic field by the nuclear spin bath in fluorine doped ZnSe, Zhukov *et al.*

SUPPLEMENTARY NOTE 1: PARAMETER SPACE OF THE MODEL

Here we provide additional calculations carried out within the theoretical model described in the main text. The average spin component S_x of the electron spin polarization is calculated by Eq. 6 of the main text. This value is substituted into the equation of the dynamic polarization of the nuclei, see Eq. 7. The solution of the equation makes it possible to obtain the Overhauser field, which is added to (or subtracted from) the external magnetic field, thereby adjusting the precession frequency of the electrons.

The result of solving the equation for the dynamic polarization of nuclei depends on the following parameters: 1) optical detuning from the absorption (trion) resonance, 2) the area of the pulse, 3) the dephasing time of one spin, 4) the type of nuclei (the hyperfine constant, the isotope content).

We first consider the dependence on laser power for a fixed value of positive and negative detuning, see Supplementary Fig. 1(a) and (b), respectively. The sign of the optical detuning Δ changes the sign of the derivative $\langle S_x(\omega_e)\rangle$, which leads to a different behavior of B_N, as shown in Supplementary Figs. 1(c) and (d). In the case of positive detunings, the maximum value of the Overhauser field is near a half-integer value of B_x/B_0. This behaviour leads to formation of plateau at an integer mode of B_x/B_0, see Supplementary Fig. 1(e). In the case of negative detunings, the maximum value of the Overhauser field is near an integer value B_x/B_0 and the plateaus are formed between the modes, Supplementary Figs. 1(d) and (f). The value of the Overhauser field has a strong dependence on the pump power and has the highest spin polarization and therefore the Overhauser field close to a π-pulse area, shown by the curves of different color in Supplementary Fig. 1. Here we do not consider the dependence of the optical detuning on the optical power. In reality (in our sample), a variation of the pump power changes the optical detuning as well, which leads to the power dependent variation of the induced Overhauser field, see Fig. 5 in the main text.

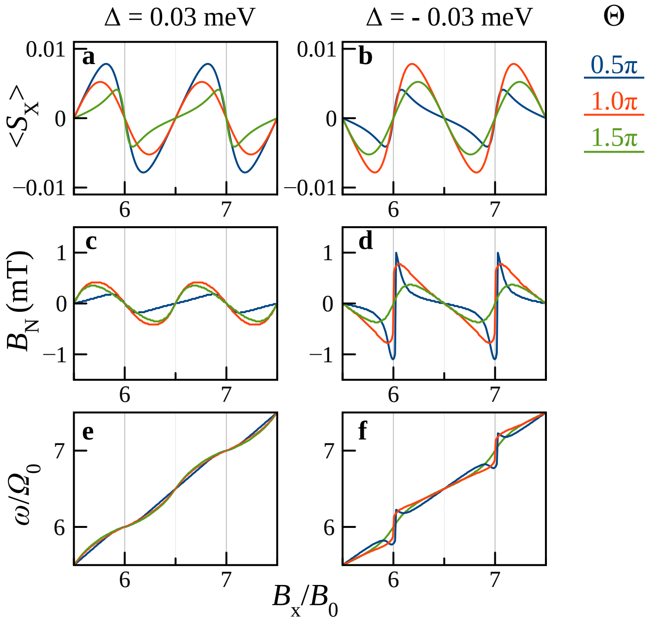

Supplementary FIG. 1. Left column: positive detuning. Right column: negative detuning. (a) and (b), gives the average spin polarization along the external magnetic field in x direction $\langle S_x(\omega_e)\rangle$; (c) and (d), gives the induced Overhauser field; (e) and (f), gives the electron precession frequency in the total field. Curves of different colors correspond to different areas of the pulse Θ (see the legend). Calculation parameters: $T_2 = 1.5T_R$, $A_{Se} = 33.6\,\mu$eV, $I_{Se} = 1/2$.

The solution of the equation for the dynamic polarization of nuclei shows three solutions in specific ranges of B_x/B_0, two of which are stable (colored points in the graph) and one unstable (dashed gray line). According to Ref. [1], the fulfillment of the following condition guarantees a stable solution of the dynamic polarization equation for the nuclei:

$$\frac{\delta\langle S_x(\omega_e + \omega_N)\rangle}{\delta\omega_N} < \frac{A\chi}{\hbar}. \tag{1}$$

If there is an unstable solution besides a stable one, a hysteresis appears, as demonstrated for negatively charged InAs quantum dots [2].

Supplementary Figure 2 demonstrates similar dependencies as Supplementary Fig. 1, but for a set of different detuning values Δ for a fixed pulse area of π. One can see that the overall amplitude behavior has a dispersive shape so that the spin polarization and Overhauser field grow for smaller detuning values and decay at higher ones. The dependencies for $\Delta = 0.32$ meV represent the detuning with the maximal spin polarization.

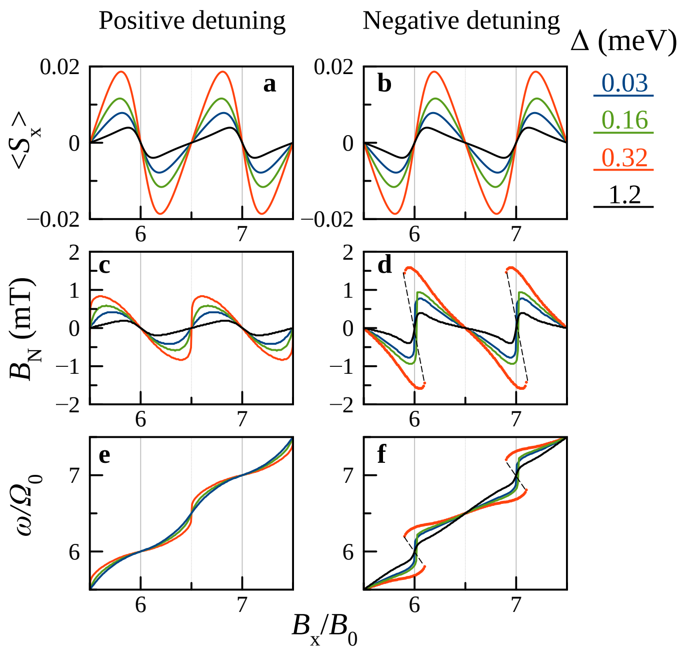

Supplementary FIG. 2. Left column: positive detuning. Right column: negative detuning. (a) and (b) gives average spin polarization $\langle S_x(\omega_e)\rangle$; (c) and (d) gives the Overhauser field; (e) and (f) gives the electron precession frequency in the total field. Calculation parameters: $\Theta = \pi$, $T_2 = 1.5T_\mathrm{R}$, $A_\mathrm{Se} = 33.6\,\mu$eV, $I_\mathrm{Se} = 1/2$. The dashed line indicates an unstable solution, the points are stable solutions.

Supplementary Figure 3 demonstrates the dependence on the spin dephasing time T_2. A longer spin dephasing time leads to a higher spin polarization and stronger Overhauser field, which in turn leads to stronger hysteresis effects.

The range of the parameters in which the hysteresis is observed is shown in Supplementary Fig. 4. Lines of different colors denote the boundaries of the region (all points above the line) of appearance of hysteresis for different types of nuclei.

For further discussion of the stability of the solutions, see Supplementary Note 5.

SUPPLEMENTARY NOTE 2: FREQUENCY DISCRETIZATION AT INCREASED MAGNETIC FIELDS

To demonstrate the step-like behavior at higher magnetic fields, we have measured the pump-probe spectra in the field range of $56 - 64$ mT. The contour plots in Supplementary Fig. 5 show a behavior very similar to the Fig. 2 in the main text. Supplementary Figure 5(c) represents additionally the calculated contour plot for the electron g-factor $g_\mathrm{e} = 1.13$ without nuclei involvement.

SUPPLEMENTARY NOTE 3: COMPARING SAMPLES WITH DIFFERENT FLUORINE CONCENTRATION

We observe a pronounced difference of the induced nuclear polarization for ZnSe:F samples with a different concentration of fluorine donors. In the sample with $n_\mathrm{D} \approx 1 \times 10^{18}\,\mathrm{cm}^{-3}$ studied in the Ref. [3] no indication of frequency plateaus is observed. On the other hand, this sample demonstrates a strongly inhomogeneous nuclear polarization around the optically detected nuclear magnetic resonances (ODNMR) defined by the helicity modulation of the pump laser f_m, which reaches values of the nuclear-induced field up to 1 mT [3]. Such NMR is also present in the current sample with $n_\mathrm{D} \approx 1 \times 10^{15}\,\mathrm{cm}^{-3}$ for ^{77}Se at $B_x = 28.87$ mT and $f_\mathrm{m} = 235$ kHz, but has a much smaller amplitude

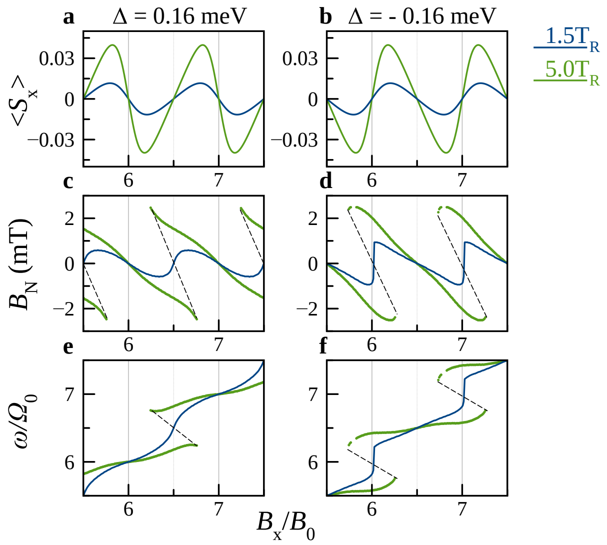

Supplementary FIG. 3. Left column: positive detuning. Right column: negative detuning. (a) and (b) gives $\langle S_x(\omega_\mathrm{e})\rangle$; (c) and (d) gives the Overhauser field; (e) and (f) gives the electron precession frequency in the total field. Curves of different colors correspond to different values of T_2 (see the legend for the values). Calculation parameters: $\Theta = \pi$, $A_\mathrm{Se} = 33.6\,\mu$eV, $I_\mathrm{Se} = 1/2$.

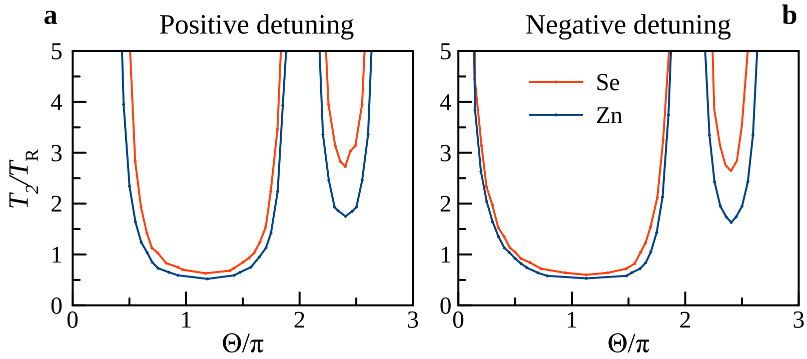

Supplementary FIG. 4. The parameter range above the lines leads to the appearance of a hysteresis for a particular type of nucleus. The values of the detunings are $\pm 0.16\,$meV, respectively. The magnitude of the magnetic field for positive detuning corresponds to an integer mode position, for negative detuning it is located between the integer modes.

on the level of 0.1 mT and is hidden by periodic magnetic field modulations of the amplitude in the range of 2 mT, see Fig. 4 in the main text. This NMR is related to the inhomogeneous nuclear polarization induced by the inhomogeneous Knight field of the donor-bound electrons. The reason for such a difference may be the proximity to the metal-insulator transition for the sample with higher donor concentration, which leads to strong fluctuations of the electrostatic potential and, as a consequence, to a strong inhomogeneity of the electron Knight field. This leads to a broad NMR and washes out the Stark-field induced oscillations with laser repetition periodicity. The sample with low donor concentration should have in comparison a much narrower NMR peak and strong oscillations of the Overhauser field.

SUPPLEMENTARY NOTE 4: TEMPERATURE DEPENDENCE OF THE INDUCED OVERHAUSER FIELD.

With increasing temperature, relaxation processes of the electron and nuclear spins become important, not related to their hyperfine interaction. Thus, the effectiveness of dynamic nuclear polarization, as a rule, decreases. Sup-

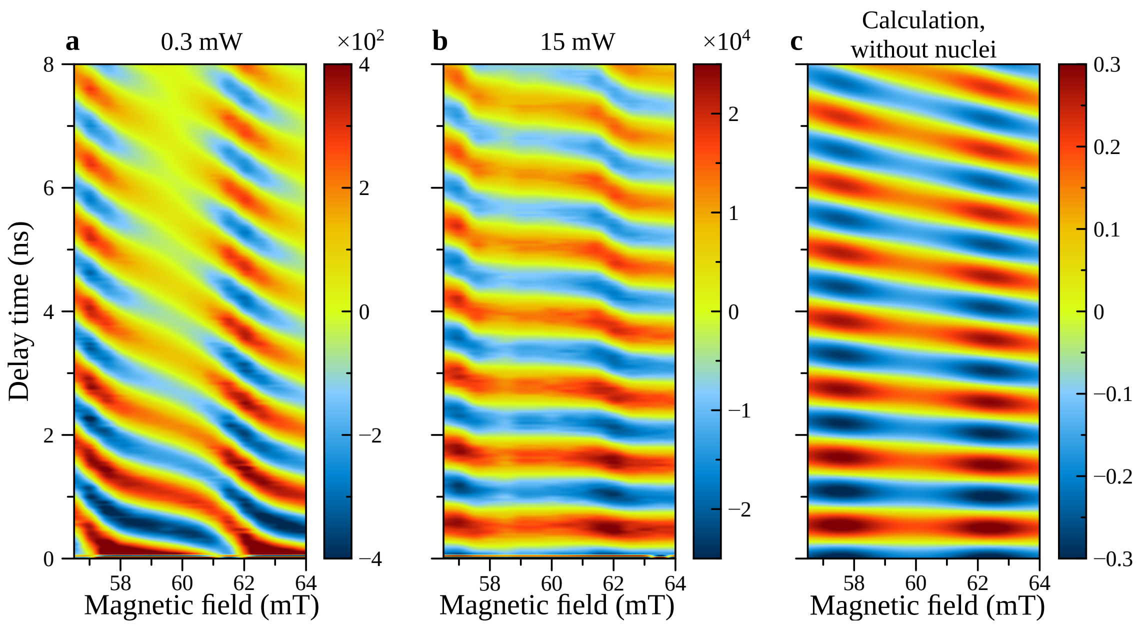

Supplementary FIG. 5. Contour plots of TRKR signal as function of external magnetic field B_x and delay time, using pump excitation powers of 0.3 mW (a) and 15 mW (b). (c) Calculated plot without nuclei involved in the process for parameters $\Theta = \pi$, $T_2 = 1.5T_\mathrm{R}$, and $\Delta = 0$.

plementary Fig. 6 provides a measurement of the induced Overhauser field at three different temperatures: 1.6, 18, 26 K. It demonstrates a slow decrease of the nuclear induced effects with rising temperature. The data is presented in relative units, as for this experiment we measured a difference of the maximal nuclear-induced field for low (0.3 mW) and high (10 mW) pump powers. Furthermore, from earlier studies of the same system (Ref. [4]) we know, that the electron spin dephasing time is constant up to 40 K, with a fast decrease at higher temperatures due to activation of the donor, which sets the upper limit of the effect.

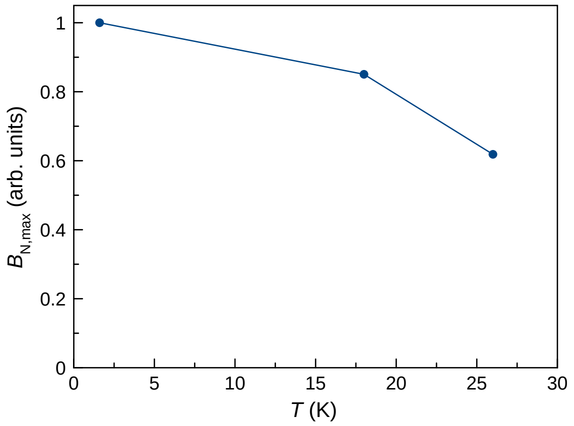

Supplementary FIG. 6. Temperature dependence of the maximally induced Overhauser field in relative units.

SUPPLEMENTARY NOTE 5: ELECTRON-NUCLEAR FEEDBACK STRENGTH AND ITS EFFECT ON ELECTRON SPIN DEPHASING

The question of the stability is solved using the dynamic equation Eq. 2 of Ref. [1] (Eq. 7 in the main text). In fact, for a small deviation of the nuclear polarization I'_N from a stationary value I_N, the equation for the dynamic

polarization of the nuclei Eq. 2 takes the form $dI'_{\rm N}/dt = -\lambda I'_{\rm N}$, where the stable state corresponds to the case of a positive Lyapunov exponent $\lambda > 0$. The larger the parameter λ, the greater the stability and the smaller the fluctuations of the nonequilibrium nuclear spin. We can regard the nuclear spin fluctuations as the result of the action of some "random force" (Langevin's approach), so that:

$$dI'_{\rm N}/dt = -\lambda I'_{\rm N} + F(t). \tag{2}$$

The average value of the random force $\langle F(t)\rangle$ is zero. We are interested in its correlation function $\langle F(t)F(t')\rangle$, where the angle brackets mean averaging over the realizations of the random force. Assuming the process to be Poissonian, we have:

$$\langle F(t)F(t')\rangle = C_I\delta(t-t'), \tag{3}$$

where the constant C_I is defined later in the text.

In our case, the characteristic square of the fluctuations of the nuclear spin is given by the equation:

$$\langle (I'_{\rm N})^2\rangle = \frac{C_I}{2\lambda}. \tag{4}$$

In the absence of a dynamic nuclear spin polarization, the parameter $|\lambda| = 1/T_1$, where T_1 is the spin-lattice relaxation time of the nuclei. In a completely disordered state $\langle (I'_{\rm N})^2\rangle = I(I+1)N$, where N is the number of nuclei in the electron localization region at the donor or in the quantum dot. Therefore, the constant $C_I = 2I(I+1)N/T_1$. In the presence of a dynamic polarization, the nuclei result in a strong feedback, and as shown in Ref. [1], the parameter λ greatly increases (by two orders of magnitude), so that $|\lambda| \gg 1/T_1$. According to Supplementary Eq. 4, this leads to a significant suppression of the nuclear spin fluctuations.

Fluctuations of the nuclear spin manifest themselves in the dephasing of the Larmor precession of electrons in an external magnetic field. The fact is, that, due to the hyperfine interaction, the nuclei create an effective magnetic field on the electrons proportional to the average spin of the nuclei $B_{\rm N} \sim I_{\rm N}$ in the donor region. From donor to donor, the nuclear fields change because of the spin fluctuations. This causes the precession of the ensemble of electrons to become out of phase, characterized by the time T_2^* due to the additional precession in a random nuclear field with a characteristic frequency $\omega_{\rm N} = \mu_{\rm B} g_{\rm e}\sqrt{\langle (B_{\rm N})^2\rangle}/\hbar$. The corresponding dephasing rate in this case is $1/T_2^* = \omega_{\rm N} \sim 1/\sqrt{|\lambda|}$. Consequently:

$$T_2^* \sim \sqrt{|\lambda|}. \tag{5}$$

Note that the specific form of the dependence Supplementary Eq. 5 is mainly determined by the character of the random process and by the explicit form of the correlation function Supplementary Eq. 3. However, the qualitative conclusion remains: the stronger the stabilizing feedback (the greater λ), the longer is the time for dephasing. The physical reason of the result is a stronger returning force (feedback) that reduces the fluctuations induced by the same force.

In Ref. [5] it is noted, that quenching of the fluctuations makes it possible to neglect them entirely and justifies the applicability of the mean-spin model in a wide range of magnetic fields. In this case, the precession frequency $\omega_{\rm N}$ in the field of unpolarized nuclei can become comparable with the distance between the modes Ω_0. Nevertheless, in the case of dynamic polarization of the nuclei with strong feedback (the plateau region), the fluctuations will be suppressed by a factor of $\sqrt{|\lambda|} \sim 10$.

SUPPLEMENTARY REFERENCES

[1] Korenev, V. L. Multiple stable states of a periodically driven electron spin in a quantum dot using circularly polarized light. *Phys. Rev. B* **83,** 235429 (2011).

[2] Kalevich, V. K., Kavokin, K. V. & Merkulov, I. A. in *Spin Physics in Semiconductors* (ed Dyakonov, M.) (Springer, Berlin, 2008).

[3] Heisterkamp, F. *et al.* Inhomogeneous nuclear spin polarization induced by helicity-modulated optical excitation of fluorine-bound electron spins in ZnSe. *Phys. Rev. B* **92,** 245441 (2015).

[4] Greilich, A. *et al.* Spin dephasing of fluorine-bound electrons in ZnSe. *Phys. Rev. B* **85,** 121303 (2012).

[5] Danon, J. & Nazarov, Y. V. Nuclear Tuning and Detuning of the Electron Spin Resonance in a Quantum Dot: Theoretical Consideration. *Phys. Rev. Lett.* **100,** 056603 (2008).
